# Usability Evaluation of a Macular Quantitative Square Grid Self-Examination Application in Patients With Macular Disease: Mixed Methods Study

**DOI:** 10.2196/79699

**Published:** 2026-03-18

**Authors:** Shu Li, Enming Zhang, Jiani Pan, Yan Xu, Kairong Zheng, Xun Xu, Qiong Fang

**Affiliations:** 1 National Clinical Research Center for Eye Diseases, Shanghai, China; 2 School of Nursing, Shanghai Jiao Tong University, No. 227 Chongqing South Road, Shanghai, China, Shanghai, 200025‌, China, 86 6384-6590, 86 6384-6590; 3 Department of Nursing, Shanghai General Hospital, Shanghai Jiao Tong University School of Medicine, Shanghai, China

**Keywords:** metamorphopsia, age-related macular degeneration, diabetic macular edema, mobile health, self-monitoring, usability evaluation, tele-ophthalmology

## Abstract

**Background:**

Digital self-monitoring applications could provide individuals with macular disease with a convenient, quantitative method for tracking metamorphopsia at home; yet the usability of such tools remains to be fully established.

**Objective:**

This study evaluated the usability of a macular quantitative square grid self-examination application, a semiquantitative, touch-based self-monitoring application for macular function.

**Methods:**

This study used a convergent mixed methods design. The application quantifies (1) distortion severity, (2) distortion area, and (3) temporal trends through a 3-step touch interface. A total of 24 adults with neovascular age-related macular degeneration or diabetic macular edema, accompanied by self-reported metamorphopsia, participated in a single supervised test session. A 10-item System Usability Scale (SUS) was used to assess usability, and semistructured interviews were conducted to gather further insights. Quantitative data were summarized descriptively, and qualitative feedback underwent inductive thematic analysis.

**Results:**

A total of 24 participants completed the GridMacuScan application self-assessment, the SUS questionnaire, and 11 participants completed the interview when data saturation was achieved. All eyes that showed distortion on the Amsler grid also produced positive distortion maps on the GridMacuScan application, yielding 100% diagnostic concordance. The mean SUS score was 82.1 (SD 8.7), indicating “good-excellent” usability. The inductive thematic analysis yielded four overarching themes: (1) high usability and positive overall experience, (2) perceived functional advantages, (3) shortcomings and optimization suggestions, and (4) strong willingness for continued use.

**Conclusions:**

The GridMacuScan application demonstrated diagnostic sensitivity comparable to that of the traditional Amsler grid and received high user ratings for usability. Furthermore, it provided quantitative distortion metrics that could facilitate longitudinal disease surveillance. Future research must be conducted to validate performance in unsupervised home environments and investigate whether sustained use improves time-to-disease-progression detection and treatment outcomes.

## Introduction

Macular diseases such as age-related macular degeneration (AMD) [1] and diabetic macular edema (DME) [2] are among the leading causes of irreversible central vision loss worldwide. A hallmark symptom of these conditions is metamorphopsia, a visual distortion resulting from structural changes in the macula [3]. Metamorphopsia has been identified as a sensitive functional biomarker of disease onset, progression, and therapeutic response [4,5]. In clinical settings, the timely detection of visual changes is particularly critical during intravitreal anti–vascular endothelial growth factor (anti-VEGF) therapy, where dynamic treatment decisions are contingent on subtle shifts in visual function [6].

However, suboptimal adherence remains a widespread challenge. A recent systematic review revealed that approximately 30% of patients discontinue anti-VEGF therapy before the prescribed duration, with dissatisfaction with treatment outcomes being the most commonly cited reason (29.9%) [7], which highlights an urgent need for tools that enhance patient engagement by making treatment responses more tangible and visible. Currently, changes in visual function are primarily determined by infrequent outpatient examinations, which exhibit limited capacity for continuous patient monitoring between visits [8].

The Amsler grid, a rudimentary paper-based tool, remains the most prevalent approach for domestic monitoring of central vision due to its affordability and accessibility [9]. However, its use in daily life remains limited, potentially due to the heterogeneity of the generated data, the inability to perform quantitative assessments, the complexity of recording processes, and the difficulty in tracking trend changes [10,11], which complicates the accurate comparison of pre- and posttreatment outcomes [12]. A meta-analysis revealed substantial variability in the sensitivity and specificity of the Amsler grid, particularly in the context of AMD screening [10]. Furthermore, traditional grids may present certain challenges in terms of usability for elderly individuals, such as maintaining central fixation and accurately reporting distortions. These limitations underscore the necessity for simplified, user-friendly tools that prioritize accessibility and consistency in real-world domestic environments [13,14].

In China, structured self-monitoring of visual function among patients with retinal disease has not been widely prioritized. Although Amsler grids are occasionally distributed in paper format, especially in community and primary care settings, they are rarely integrated into standardized follow-up workflows or supported with user training [15]. This finding indicates a systemic deficiency in awareness, patient empowerment, and digital tool adoption in the management of chronic retinal disease [15].

In recent years, digital health innovations have introduced app-based tools to support remote self-monitoring of visual function [16]. Although applications such as MacuFix [17], OdySight [18], and Home Vision Monitor [19] have demonstrated efficacy in Europe and the United States, their implementation in China remains constrained by numerous barriers. For instance, the MacuFix application requires users to discriminate among 4 simultaneously presented visual fields to identify subtle distortions. This interface design presents potential cognitive and operational challenges for older adults with visual impairments [17]. In addition, the majority of these applications are deficient in localization of language, user interface design, and integration with domestic health care systems and platforms [20].

To address these challenges, we developed GridMacuScan, an application compatible with tablet devices, designed for structured, self-administered metamorphopsia assessment using a digital square grid. The name “GridMacuScan” originates from the traditional Amsler grid pattern, while incorporating the terms “macula” and “scan” to emphasize its focus on systematically monitoring and quantifying macular degeneration. The GridMacuScan application enables patients to visualize and quantify visual distortion before and after anti-VEGF treatment, providing real-time data to inform clinician decision-making and support patient self-management.

The aim of this study was to evaluate the usability and acceptability of the GridMacuScan application among individuals with macular disease using a mixed methods approach and to characterize user experience, identify potential barriers to engagement, and generate insights to inform future refinement and clinical integration.

## Methods

### Overview

This study followed the IDEAS (Integrate, Design, Assess, and Share) framework for developing digital health behavior change interventions [21]. According to the IDEAS framework, rigorous evaluations of digital products through large-scale randomized controlled trials should be preceded by small-scale evaluations to test potential efficacy and usability. Specifically, a questionnaire can be used to evaluate usability and satisfaction, and interviews can be conducted to gain insight into the user experiences.

### Study Design

This study employed a convergent mixed methods design to evaluate the usability of the GridMacuScan application. Quantitative data were collected through a standardized survey, and qualitative insights were gathered through semistructured interviews, enabling a comprehensive assessment of the user experience. The convergent design involves the parallel collection and analysis of both types of data, subsequently merged to yield a more thorough understanding than that afforded by either method alone. This approach was selected over other mixed methods designs (eg, explanatory, exploratory, or embedded) because it allows for simultaneous triangulation of findings, balancing the strengths and limitations of each data type [22]. The study was conducted in two phases: (1) the development of the GridMacuScan application tailored for patients with macular disease and (2) a usability evaluation combining quantitative assessment and qualitative feedback.

### Participation Recruitment and Eligibility Assessment

Participants were recruited from the ophthalmology outpatient department of Shanghai General Hospital between March 4 and March 22, 2025. The inclusion criteria were as follows: (1) aged 18 years or older; (2) diagnosed during outpatient consultation with AMD or DME in at least 1 eye; (3) presence of metamorphopsia, confirmed using the Amsler grid test; (4) best-corrected visual acuity ≥20/200 in the affected eye; and (5) ability to understand study instructions and complete the assessment with minimal assistance. Exclusion criteria included (1) diagnosis of advanced glaucoma, retinal detachment, or other ocular conditions known to cause metamorphopsia or interfere with central visual field assessment; (2) presence of severe cognitive impairment, motor disability, or other systemic diseases that may hinder independent operation; and (3) presence of bilateral macular disease requiring immediate intervention, which could confound the usability evaluation. For the sample size, Bastien [23] cited studies showing that the majority of usability problems can be identified in a sample of 5 to 15 participants. Assuming a 20% dropout rate for the clinical sample, it was calculated that a minimum of 18 participants would need to be invited to participate in the study.

### Development of the GridMacuScan Application

The GridMacuScan application was developed as a web-based self-monitoring tool tailored for individuals with macular disease. The name “GridMacuScan” reflects the application’s 3 core components: grid-based visualization, macular disease monitoring, and scanning for distortion. The tool was designed to enable structured, semiquantitative self-assessment of metamorphopsia via a square digital grid, providing both patients and clinicians with meaningful visual feedback over time.

This application is composed of 2 primary modules: basic information collection and dynamic disease monitoring. The basic information module is designed to collect user data, including systemic comorbidities, educational background, and other relevant demographic characteristics, which may inform the feasibility of continued follow-up and digital tool use. The dynamic monitoring module is designed to record patient-reported visual distortions, enabling the quantification of metamorphopsia changes before and after intravitreal anti-VEGF treatment.

This application also incorporates a backend management platform encompassing 2 core functionalities: data management and disease monitoring. The data module aggregates longitudinal self-assessment results to generate metamorphopsia trend profiles automatically. The monitoring module enables authorized clinical personnel (eg, retinal specialists) or system administrators to review patient-level progression patterns and intervene as deemed necessary.

### Self-Assessment Workflow and User Interface Design

**Figure 1.**
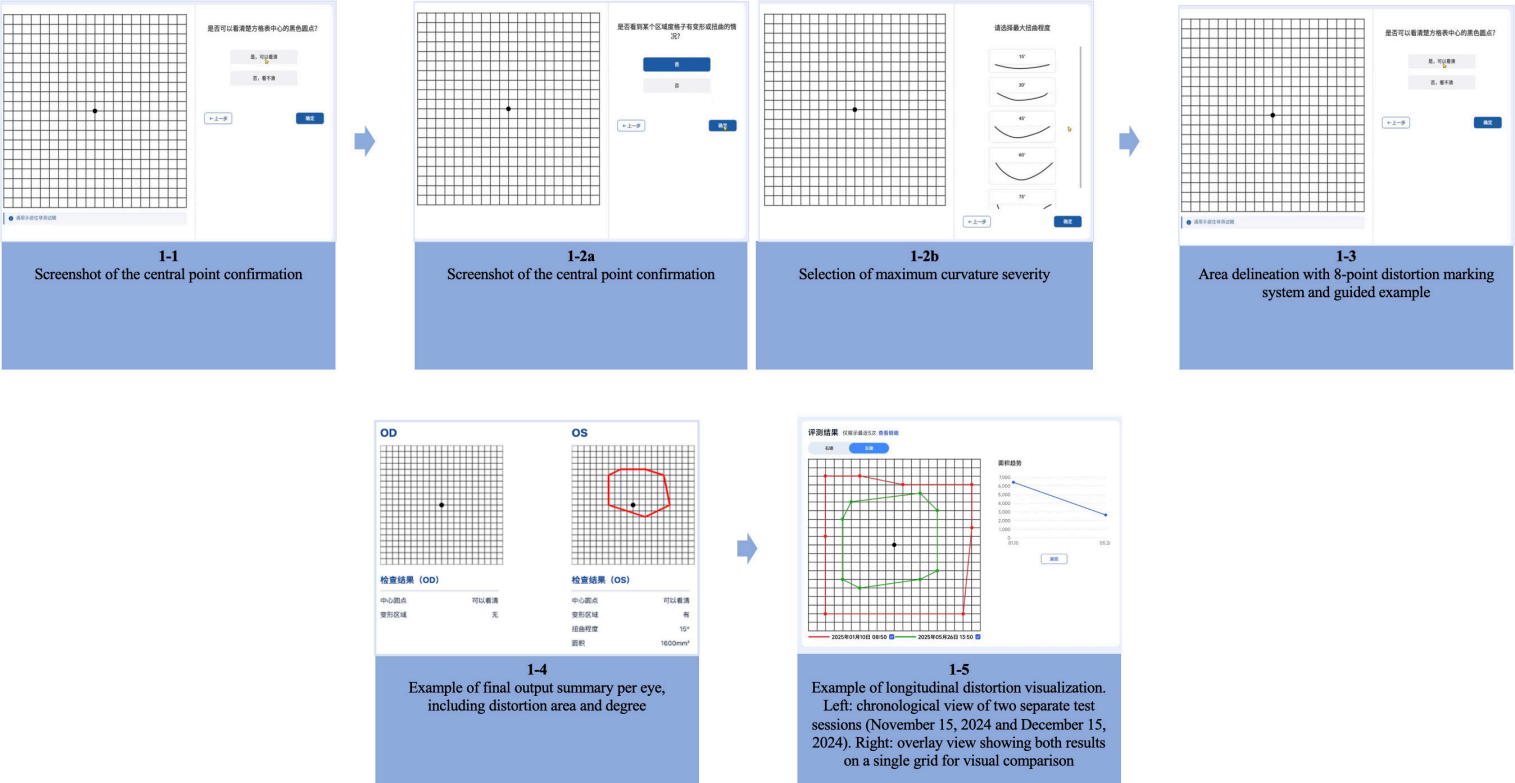
Self-assessment workflow of the GridMacuScan application.

The GridMacuScan application’s self-assessment workflow comprises the following 3 sequential submodules:

- Step 1: Central point confirmation—Users are prompted to focus on a central black dot displayed within a digital grid and indicate whether it appears clear, blurry, or distorted. This step assesses central fixation stability and initiates the evaluation process (Figure 1).

- Step 2: Severity selection—Users are required to confirm the presence of metamorphopsia (Step 2a in Figure 1). Following this confirmation, they are required to select the severity by choosing from schematic illustrations representing varying degrees of curvature (15° to 75°; Figure 1).
- Step 3: Area delineation—Users must mark the approximate spatial extent of the distortion by selecting grid cells corresponding to the affected area. A rule-based selection strategy instructs users to identify and mark the outermost boundaries of distortion along 4 cardinal directions (superior, inferior, nasal, and temporal), resulting in 8 structured anchor points. A dynamic progress bar (eg, “3/8 points completed”) and illustrative prompts were provided to enhance clarity and task completion, based on the standardized 8-point rule, which requires users to mark the outermost distorted grid lines in 4 directions (Step 3 in Figure 1).

Regarding the interface design, the GridMacuScan application prioritizes visual accessibility and ease of use. It is acknowledged that a large number of individuals with vision loss may encounter difficulties in accessing digital technologies. To address this issue, the user interface has been meticulously designed to incorporate large icons, high-contrast color schemes, and low-cognitive-load layouts. These features have been adapted for the use of older adults and individuals with low vision.

### Technical Implementation and Data Visualization

From a technical standpoint, the front end was developed using Taro, a cross-platform framework based on HTML and JavaScript, ensuring compatibility across devices. The back end was implemented in Java to enhance data stability and processing efficiency. The tool was developed to be compatible with commonly available Android tablets. It was tested on an 11-inch Redmi Pad SE (Xiaomi Corporation) device designed to prioritize a basic user experience and exceptional value for money, which was selected based on its affordability, screen size, and suitability for domestic use.

In the result visualization module, the GridMacuScan application supports a multilevel display of distortion changes. After each self-assessment, the results are stored in the patient record and visualized as a quantifiable grid. Users can access their historical records and review prior results in either a chronological or layered overlay mode (Step 4 in Figure 1). The system provides date-stamped output with area and curvature comparisons, enabling users and clinicians to observe trends in metamorphopsia severity over time. In the overlay view, changes in the distortion area are visualized through color-coded outlines and anchor points, with the area growth or reduction ratios being calculated automatically to support clinical interpretation and patient engagement (Step 5 in Figure 1).

To preserve the diagnostic logic of conventional tools, the GridMacuScan application adopted a one-to-one digital replication of the traditional Amsler grid layout, as prior studies demonstrated no significant performance differences across grid densities used in electronic Amsler tests [24]. An illustration of the GridMacuScan application output interface is shown in Steps 4 and 5 in Figure 1, which display monocular detection results, including fixation clarity, distortion presence, angular distortion, and area measurement.

### Procedures

To ensure the stable operation and implementation of the GridMacuScan application, a backend management team was established. This team comprised 2 research assistants, 2 ophthalmologists, and 1 software engineer. The research assistants were responsible for participant recruitment, daily user management, and backend data organization. Their duties included monitoring the usage status of all registered users, managing login activity, organizing and backing up usage data, and serving as the primary point of contact for participants encountering any operational issues while using the GridMacuScan application. The ophthalmologists conducted a clinical evaluation, with a particular focus on interpreting optical coherence tomography (OCT) images and comparing structural findings with functional outcomes captured by the GridMacuScan application. The software engineer was responsible for maintaining the technical functionality of the application, including backend monitoring of coding or logic errors, resolving system faults promptly, and performing routine updates to ensure long-term operability. The usability evaluation was conducted by the research assistants after the testing period, using both a standardized questionnaire and semistructured interviews to assess user experience and gather feedback for future refinement.

### Data Collection

#### Testing Preparation and Participant Training

Before the initiation of the usability testing phase, the participants’ baseline demographic and clinical information was collected, including age, sex, education level, ocular diagnosis, and current use of intravitreal medication. These data were then used to describe the sample characteristics and assess the generalizability of the study findings. Each participant was provided with a standardized user guide and underwent a concise, in-person training session conducted by the research staff. The training program incorporated step-by-step demonstrations using illustrative examples to familiarize users with the functions and operations of the GridMacuScan application. Participants were then invited to complete the full self-assessment procedure independently with minimal supervision.

#### Quantitative Evaluation of the Usability of the GridMacuScan Application

After completing the test, the System Usability Scale (SUS) is used to quantitatively assess the usability of the GridMacuScan application. All responses were collected anonymously in paper format. SUS is a widely adopted 10-item instrument for assessing the perceived usability of digital health tools [25]. The SUS comprises alternating positive and negative statements, each of which is rated on a 5-point Likert scale ranging from “strongly disagree” to “strongly agree.” The individual item scores were transformed and summed by standard procedures and then multiplied by 2.5 to yield a total score ranging from 0 to 100, with higher scores indicating better usability.

#### Qualitative Assessment of the User Experience With the GridMacuScan Application

A semistructured interview was conducted to complement the quantitative assessment and to gain deeper insights into users’ experiences with the GridMacuScan application. Each interview was conducted by a researcher who had undergone training in qualitative methods and lasted approximately 30 minutes. The interview process continued until data saturation was achieved, defined as the point at which no new themes emerged from 2 consecutive interviews. It is important to note that all interviews were audio-recorded and transcribed verbatim for thematic analysis. The interview guide was composed of three key questions: (1) What do you think are the main advantages of this self-monitoring tool? How does it help you manage your condition? (2) What limitations or weaknesses did you find? Do you have any suggestions on how to improve it? (3) Would you be willing to keep using this application to monitor your condition? Why or why not?

### Data Analysis

#### Quantitative Evaluation Data of the Usability of the GridMacuScan Application

Descriptive statistics were used to summarize the participants’ sociodemographic and clinical characteristics as well as their SUS scores. Categorical variables, including sex and education level, were reported as frequencies and percentages. Continuous variables, including age and SUS scores, were presented as means and SDs. The analysis of the quantitative data was conducted using the R Studio software (version 2024.4.2.764; R Foundation for Statistical Computing).

#### Qualitative Assessment Results of the User Experience With the GridMacuScan Application

Following each interview, the interviewer completed the review of the audio recordings within 24 hours, completed verbatim transcription, and imported the transcripts into NVivo 14.0 (QSR International) for analysis. The transcripts were analyzed systematically using the inductive thematic analysis as described by Braun and Clarke [26]. Two researchers independently reviewed the transcripts multiple times to become thoroughly acquainted with the content. Subsequently, they engaged in open coding to identify significant statements and formulate meanings. The resulting codes were meticulously cross-checked, and any discrepancies were discussed and resolved through a consensus-based approach. The analogous codes were then grouped into subthemes, which were subsequently organized under broader themes that reflected the core dimensions of user experience. A series of illustrative quotations was selected to provide a supporting framework for the themes and subthemes that were identified.

### Mixed Methods Integration

A convergent mixed methods approach was used to integrate and interpret the quantitative and qualitative findings [22]. The integration process comprised four distinct steps: (1) analyzing the quantitative data, (2) analyzing the qualitative data, (3) identifying areas of similarity or divergence, and (4) interpreting whether the findings confirmed, expanded, or contradicted each other. Confirmation was established when both data types exhibited analogous usability outcomes. The expansion resulted from the 2 data sources providing divergent yet complementary insights into the user experience. The discordance was identified when the survey and interview findings were inconsistent or contradictory, prompting further interpretive analysis.

### Ethical Considerations

The ethical approval for the study protocol was obtained from the Ethics Committees of Shanghai General Hospital, Shanghai Jiao Tong University School of Medicine (2022-KY-021). Before the study began, all participants provided written consent by signing an informed consent form. The participants were told that their participation in the study was voluntary, that they could withdraw at any time, and that all data would be kept strictly confidential and would only be accessible to the researchers. No monetary or material compensation was provided to the participants.

## Results

### Participants’ Characteristics

A total of 24 participants were recruited from the ophthalmology outpatient department of Shanghai General Hospital between March 4 and March 22, 2025. All 24 participants completed the GridMacuScan application self-assessment and the SUS questionnaire. All 24 participants were also required to complete semistructured interviews.

The mean age of the participants was 60.2 (SD 13.9) years. The total number of participants in the study was 24, comprising 13 (54%) men and 11 (46%) women. Regarding education, 13 (54%) participants had attained college or university-level education. The employment status of the participants was evenly distributed, with 12 (50%) participants being employed and 12 (50%) retired. The majority of the participants were diagnosed with AMD (n=14, 58%), while 10 (42%) had DME. The mean number of intravitreal anti-VEGF injections received by participants was 3.5 (SD 1.8), with a median of 3 (IQR 2‐5). A comprehensive overview of the demographic and clinical characteristics is provided in Table 1.

**Table 1.** Demographic characteristics of participants (N=24).

Characteristic	Value
Age (y), mean (SD)	60.2 (13.9)
Gender, n (%)	
Male	13 (54)
Female	11 (46)
Education, n (%)	
Primary school and below	2 (8)
Junior high school	2 (8)
Senior high school	7 (29)
College or university and above	13 (54)
Occupation status, n (%)	
Employed	12 (50)
Retired	12 (50)
Type of disease, n (%)	
AMD^a^	14 (58)
DME^b^	10 (42)
Injection eye, n (%)	
Left eye only	7 (29)
Right eye only	11 (46)
Both eyes	6 (25)
Medication, n (%)	
Aflibercept	3 (12)
Conbercept	12 (50)
Ranibizumab	9 (38)
Number of injections, median (IQR)	3 (2-5)

a AMD: age-related macular degeneration.

b DME: diabetic macular edema.

### Quantitative Evaluation of the Usability of the GridMacuScan Application

#### Diagnostic Agreement With the Amsler Grid

Before the usability testing, all participants had been confirmed to have metamorphopsia based on traditional Amsler grid screening as per the inclusion criteria. The GridMacuScan application detected visual distortion in all 24 cases, resulting in 100% concordance with the traditional Amsler grid findings for the presence of self-reported metamorphopsia.

#### SUS Scores

The overall mean SUS score for GridMacuScan application was 82.08 (SD 8.68), indicating “excellent” usability according to established benchmarks (28). Item-level analysis revealed generally positive responses. For illustration, 96% (23/24) of the participants expressed an agreement or strong agreement with the statement “I found the test easy to use” (item 3). Conversely, for negatively framed items, participants indicated high usability. For instance, for the item “I found the test unnecessarily complicated” (item 2), the mean score was 3.50 (SD 0.83) on a scale where lower scores (disagreement) indicate better usability after reverse scoring. A parallel outcome was observed for the statement “I had to learn a lot before using the test” (item 10), for which the mean score was 3.71 (SD 0.46). None of the participants reported that the system was found to be overly complicated (item 8) or that extensive prior learning was required before its usage (item 10). Table 2 presents the mean scores and SDs for all 10 SUS items.

**Table 2.** Mean scores and SDs of System Usability Scale (SUS) items.

Item number	SUS item statement	Mean (SD)
1	I can well imagine using the test regularly (P)	3.16 (0.48)
2	I find the test unnecessarily complicated (N)	3.50 (0.83)
3	I find the test easy to use (P)	3.13 (0.45)
4	I think I would need technical support to use the test (N)	2.21 (0.78)
5	I find that the various functions are easy to use (P)	3.46 (0.59)
6	I think there are too many inconsistencies (N)	3.71 (0.46)
7	Most people can learn to use it quickly (P)	3.33 (0.56)
8	I find the operation very complicated (N)	3.38 (0.50)
9	I felt very confident in using the test (P)	3.25 (0.53)
10	I had to learn a lot before using the test (N)	3.71 (0.46)

#### Task Completion Time

The majority of sessions lasting less than 15 minutes entailed single-eye assessments, whereas those exceeding 20 minutes frequently involved bilateral testing.

### Qualitative Assessment of the User Experience With the GridMacuScan Application

Data saturation was achieved after conducting 11 consecutive interviews with participants. The detailed characteristics of the participants are shown in Multimedia Appendix 1.

The thematic analysis of the interview data yielded 4 main themes and 7 subthemes regarding user experience with GridMacuScan. The primary themes that emerged from this analysis were as follows: (1) overall positive experience and ease of use, (2) perceived advantages of GridMacuScan, and (3) shortcomings and optimization suggestions. A detailed summary of themes, subthemes, and illustrative participant quotes is presented in Table S2 in Multimedia Appendix 2.

The participants’ reports indicated a predominantly positive experience, with frequent emphasis on highlighting the tool’s ease of use. Several key advantages were identified, including the ability to dynamically evaluate and continuously track visual distortion, the convenience of the tool, and an increased sense of confidence in their treatment. The suggestions for improvement primarily focused on developing a WeChat mini-program version to enhance accessibility and optimizing certain module functions, such as providing more detailed feedback on the degree of distortion. The majority of participants also expressed a strong willingness to continue using the GridMacuScan application for self-monitoring.

### Mixed Methods Integration

Furthermore, the qualitative findings provided a more in-depth perspective on the specific aspects that contributed to the positive usability scores. For instance, while SUS item 3 (“I find the test easy to use”) demonstrated a high score, interview data revealed that features such as a “clear and concise interface” contributed to this perception (Patient 1 quote). Similarly, the mean task completion time was 12.3 (SD 4.96) minutes, indicating the practicality of the proposed application, which was corroborated by qualitative comments on the convenience of the tool.

The quantitative finding that 23 (95.8%) participants would “imagine using the test regularly” (SUS item 1, if this is the interpretation of that item’s score from Table 2) was strongly echoed in the qualitative interviews, where a dedicated theme “Hope to continue using” emerged, with participants expressing a clear willingness to integrate the GridMacuScan application into their routine monitoring.

Qualitative feedback also identified areas for future improvement that were not explicitly captured by the SUS, such as the desire for a WeChat mini-program and enhanced analytical features. These suggestions offer valuable insights for future improvements to GridMacuScan, even with its current high usability scores. In general, there was no significant discordance observed between the quantitative and qualitative results. Indeed, the qualitative data effectively served to confirm and expand upon the quantitative usability assessment.

## Discussion

### Principal Results

The main findings of this study were that the GridMacuScan application demonstrated excellent usability, as evidenced by a high mean SUS score of 82.08 (SD 8.68), and the quantitative result was strongly corroborated by our qualitative findings, where “High usability and positive overall experience” emerged as a dominant theme. The participants frequently described the tool as “simple and intuitive’” and “easy to use.” In addition, the tool demonstrated 100% concordance with the traditional Amsler grid in detecting the presence of metamorphopsia and was completed within a feasible timeframe, with a mean completion time of 12.3 (SD 4.96) minutes.

Although traditional self-monitoring tools, such as the Amsler grid, have been employed in macular disease care, digital health innovations offer novel opportunities to overcome the inherent limitations of static, paper-based methods [27]. With the increasing use of anti-VEGF therapies for conditions such as neovascular AMD and DME, the clinical emphasis has shifted toward long-term monitoring and adherence [28,29]. In this context, the GridMacuScan application signifies a substantial advancement in patient-centered monitoring. Through its intuitive digital interface and standardized 3-step process, it enables the structured, quantifiable, and systematic capture of distortion severity and spatial extent, making it suitable for remote use and serial tracking [6]. The integration of a patient-centered interface with quantitative output effectively addresses the key limitations of paper-based grids, which are often underused in clinical practice due to their noninteractive nature and limited ability to support disease monitoring [17,30].

The design of the GridMacuScan application was guided by the principle of maximizing usability for home-based, semiquantitative monitoring, particularly among older adults. Consequently, we deliberately avoided incorporating features such as eye-tracking or fixation correction, as the added complexity could compromise adoption and ease of use. During design discussions, the tool’s visual grid structure and rule-based distortion marking were deemed sufficient for detecting meaningful changes in perceived distortion. This focus on accessible self-monitoring underscores the tool’s intended role: it is not meant to replace clinical imaging modalities, such as OCT, but to complement them by supporting regular patient engagement and symptom tracking between visits.

The GridMacuScan application shows significant promise as a tool to augment standard care for patients with macular disease. Its high usability suggests that it could be feasibly integrated into clinical workflows to empower patients with structured self-monitoring, bridge the monitoring gap between clinic appointments, and improve communication and shared decision-making. The backend platform, which aggregates data for clinician review, provides a direct pathway for integrating patient-generated health data into treatment plans.

A primary strength of this study is its rigorous mixed methods convergent design. By integrating quantitative SUS data with rich qualitative insights from interviews, we achieved triangulation of our findings, providing a more robust and comprehensive understanding of the user experience than either method could alone. Furthermore, the study was conducted with the target clinical population—patients with confirmed AMD and DME recruited from an ophthalmology outpatient department—enhancing the real-world relevance of our findings.

However, this study still has several limitations that need to be acknowledged. First, the sample size, although appropriate for identifying key usability issues (N=24), is relatively small and may limit the generalizability of the findings. Second, the participants were recruited from a single, large academic hospital in Shanghai, and a high proportion (n=13, 54%) had a college-level education or higher. This population may not be representative of patients in community or rural settings, who may exhibit different levels of digital literacy. Finally, the study focused on short-term usability rather than long-term utility, which is a key limitation and a crucial aspect that should be considered in future research. Although it was determined that users could operate the tool effectively during an initial encounter, the investigation did not extend to examining whether they would maintain engagement and adhere to regular use over time. Consequently, the ultimate clinical efficacy of the GridMacuScan application, whether its sustained usage results in discernible benefits, such as the earlier detection of disease progression or improved treatment outcomes, remains unmeasured.

To address the study’s limitations and build upon its potential, future research should focus on the following key fronts. First, the tool’s real-world performance and quantitative outputs must undergo rigorous validation. This entails conducting both unsupervised, home-based studies and longitudinal research to correlate GridMacuScan metrics with objective OCT findings. Second, establishing clinical efficacy is of the utmost importance. A large, multicenter randomized controlled trial is required to rigorously measure long-term adherence and its ultimate impact on clinical outcomes, such as earlier detection of disease progression and changes in treatment frequency. Finally, to ensure long-term values and scalability, implementation research should investigate ways to integrate the GridMacuScan application into existing telemedicine platforms and clinical workflows, thereby ensuring its utility across larger and more diverse patient populations.

### Conclusions

This mixed methods study found that the GridMacuScan application is a highly usable and acceptable tool for self-monitoring of metamorphopsia among patients with macular disease in China. It effectively addresses the known limitations of the traditional Amsler grid by providing a quantitative, trackable, and engaging user experience. However, further validation is required to ascertain its long-term adherence and clinical efficacy in a larger trial. The GridMacuScan application is a promising digital health solution with the potential to enhance patient self-management, improve engagement with therapy, and ultimately, support better clinical outcomes in the management of chronic retinal conditions.

## Supplementary material

10.2196/79699 Multimedia Appendix 1 Demographic characteristics of participants interviewed (N=11).

10.2196/79699 Multimedia Appendix 2 Thematic analysis of participant interviews regarding Gridmacuscan application usability (N=11).

## Abbreviations

AMD: age-related macular degeneration
anti-VEGF: anti–vascular endothelial growth factor
DME: diabetic macular edema
IDEAS: Integrate, Design, Assess, and Share
OCT: optical coherence tomography
SUS: System Usability Scale

## References

[1] Thomas CJ, Mirza RG, Gill MK (2021). Age-related macular degeneration. Med Clin North Am.

[2] Zhang J, Zhang J, Zhang C (2022). Diabetic macular edema: current understanding, molecular mechanisms and therapeutic implications. Cells.

[3] Wiecek E, Lashkari K, Dakin SC, Bex P (2015). A statistical analysis of metamorphopsia in 7106 amsler grids. Ophthalmology.

[4] Wiecek E, Lashkari K, Dakin SC, Bex P (2014). Novel quantitative assessment of metamorphopsia in maculopathy. Invest Ophthalmol Vis Sci.

[5] Hanumunthadu D, Lescrauwaet B, Jaffe M (2021). Clinical update on metamorphopsia: epidemiology, diagnosis and imaging. Curr Eye Res.

[6] Chew EY, Clemons TE, Bressler SB, AREDS2-HOME Study Research Group (2014). Randomized trial of a HOME monitoring system for early detection of choroidal neovascularization HOME monitoring of the Eye (HOME) study. Ophthalmology.

[7] Shahzad H, Mahmood S, McGee S (2023). Non-adherence and non-persistence to intravitreal anti-vascular endothelial growth factor (anti-VEGF) therapy: a systematic review and meta-analysis. Syst Rev.

[8] Faes L, Bachmann LM, Sim DA (2020). Home monitoring as a useful extension of modern tele-ophthalmology. Eye (Lond).

[9] Amsler M (1947). L’Examen qualitatif de la fonction maculaire. Ophthalmologica.

[10] Faes L, Bodmer NS, Bachmann LM, Thiel MA, Schmid MK (2014). Diagnostic accuracy of the Amsler grid and the preferential hyperacuity perimetry in the screening of patients with age-related macular degeneration: systematic review and meta-analysis. Eye (Lond).

[11] Bjerager J, Schneider M, Potapenko I (2023). Diagnostic accuracy of the Amsler grid test for detecting neovascular age-related macular degeneration: a systematic review and meta-analysis. JAMA Ophthalmol.

[12] Gross N, Bachmann LM, Islam M (2021). Visual outcomes and treatment adherence of patients with macular pathology using a mobile hyperacuity home-monitoring app: a matched-pair analysis. BMJ Open.

[13] Schuchard RA (1993). Validity and interpretation of Amsler grid reports. Arch Ophthalmol.

[14] Liu L, Wang YZ, Bedell HE (2014). Visual-function tests for self-monitoring of age-related macular degeneration. Optom Vis Sci.

[15] Balaskas K, Drawnel F, Khanani AM, Knox PC, Mavromaras G, Wang YZ (2023). Home vision monitoring in patients with maculopathy: current and future options for digital technologies. Eye (Lond).

[16] Daich Varela M, Sanders Villa A, Pontikos N, Crossland MD, Michaelides M (2025). Digital health and wearable devices for retinal disease monitoring. Graefes Arch Clin Exp Ophthalmol.

[17] Claessens D, Ichhpujani P, Singh RB (2022). MacuFix® versus Amsler grid for metamorphopsia categorization for macular diseases. Int Ophthalmol.

[18] Brucker J, Bhatia V, Sahel JA, Girmens JF, Mohand-Saïd S (2019). Odysight: a mobile medical application designed for remote monitoring—a prospective study comparison with standard clinical eye tests. Ophthalmol Ther.

[19] Korot E, Pontikos N, Drawnel FM (2022). Enablers and barriers to deployment of smartphone-based home vision monitoring in clinical practice settings. JAMA Ophthalmol.

[20] Kreienbrinck A, Hanft-Robert S, Forray AI, Nozewu A, Mösko M (2025). Usability of technological tools to overcome language barriers in healthcare- a scoping review. Arch Public Health.

[21] Mummah SA, Robinson TN, King AC, Gardner CD, Sutton S (2016). IDEAS (integrate, design, assess, and share): a framework and toolkit of strategies for the development of more effective digital interventions to change health behavior. J Med Internet Res.

[22] Robinson P (2007). Designing and conducting mixed methods research. Aust N Z J Public Health.

[23] Bastien JMC (2010). Usability testing: a review of some methodological and technical aspects of the method. Int J Med Inform.

[24] Augustin AJ, Offermann I, Lutz J, Schmidt-Erfurth U, Tornambe P (2005). Comparison of the original Amsler grid with the modified Amsler grid: result for patients with age-related macular degeneration. Retina.

[25] Lewis JR (2018). The system usability scale: past, present, and future. Int J Hum Comput Interact.

[26] Braun V, Clarke V (2006). Using thematic analysis in psychology. Qual Res Psychol.

[27] Marmor MF (2000). A brief history of macular grids: from Thomas Reid to Edvard Munch and Marc Amsler. Surv Ophthalmol.

[28] The Diabetic Retinopathy Clinical Research Network (2015). Aflibercept, bevacizumab, or ranibizumab for diabetic macular edema. N Engl J Med.

[29] Angermann R, Hofer M, Huber AL (2022). The impact of compliance among patients with diabetic macular oedema treated with intravitreal aflibercept: a 48-month follow-up study. Acta Ophthalmol.

[30] Crossland M, Rubin G (2007). The Amsler chart: absence of evidence is not evidence of absence. Br J Ophthalmol.

