# Supplementary material for: Usability Evaluation of a Macular Quantitative Square Grid Self-Examination Application in Patients With Macular Disease: Mixed Methods Study
Source: JMIR Hum Factors. 2026 Mar 18;13:e79699. doi: 10.2196/79699 (PMC12998605; doi:10.2196/79699)
Supplement: Multimedia Appendix 1 [file humanfactors-v13-e79699-s001.docx]

| **﻿Supplementary Table 1 Demographic characteristics of participants interviewed (N=11)** | |
| --- | --- |
| **Characteristic** | **N (%)** |
| **Age** (Mean ± SD) | 59.8 ± 8.2 |
| **Gender** |  |
| Male | 8 (73) |
| Female | 3 (27) |
| **Education** |  |
| Primary school and below | 1 (9) |
| Junior high school | 2 (1) |
| Senior high school | 4 (36) |
| College or university and above | 4 (36) |
| **Occupation status** |  |
| Employed | 4 (36) |
| Retired | 7 (64) |
| **Type of Disease** |  |
| AMD | 7 (64) |
| DME | 4 (36) |
| **Injection Eye** |  |
| Left Eye | 5 (46) |
| Right Eye | 4 (36) |
| Both Eyes | 2 (18) |
| **Medication** |  |
| Conbercept | 6 (55) |
| Ranibizumab | 5 (45) |
| **Number of Injections (Median, IQR)** | 2 (1-4) |
