# Supplementary material for: Usability Evaluation of a Macular Quantitative Square Grid Self-Examination Application in Patients With Macular Disease: Mixed Methods Study
Source: JMIR Hum Factors. 2026 Mar 18;13:e79699. doi: 10.2196/79699 (PMC12998605; doi:10.2196/79699)
Supplement: Multimedia Appendix 2 [file humanfactors-v13-e79699-s002.docx]

| **﻿Supplementary Table 2** Thematic analysis of participant interviews regarding Gridmacuscan application usability (n=11) | | | |
| --- | --- | --- | --- |
| **Theme** | **Subtheme** | **Author’s summary** | **Participants’ Quotes** |
| **1. High Usability and Positive Overall Experience** | 1.1 Simple and Intuitive Operation | Patients have reported that the advantage of this self-monitoring software was its ease of use. | “The overall feeling is quite good. It's very easy to use on the computer and the interface is clear and concise after logging into the system.” (Patient 1) |
|  |  |  | “It's easy to use and simple to operate.” (Patient 5) |
|  |  |  | “It is more convenient to operate the monitoring tool.” (Patient 12) |
| **2. Core Advantages of GridMacuScan** | 2.1 Enables Dynamic Evaluation and Continuous Tracking | Patients thought this monitoring tool was a way to record how their vision distortion changed before and after the injection. | “This self-monitoring tool let me check my visual distortion every day, which I hadn’t been able to judge properly in the past.” (Patient 3) |
|  |  |  | “I think the software is excellent. It provides a quantitative record of the changes in vision distortion before and after my injections and analyses the trends.” (Patient 14) |
|  | 2.2 Enhancing confidence in treatment | Patients expressed that the use of the GridMacuScan application could enhance confidence in anti-VEGF treatment. | “I feel an improvement in my confidence in treatment. This tool gave me a way of monitoring changes in condition, which made me more confident.” (Patient 4) |
| **3. Shortcomings and Optimization Suggestions** | 3.1 Desire for WeChat Mini-Program Development | Patients are currently engaged with the application on the device, and the proposal is for the user to transfer their activity to the WeChat application. | “The tool for monitoring is currently on a pad, which is a bit of a pain, so I'm hoping we can do this using a WeChat mini program in the future.” (Patient 2) |
|  |  |  | “You'll need a computer or a PAD for monitoring, unlike in a WeChat small program, which may be more convenient to use.” (Patient 13) |
|  | 3.2 Need for Further Optimization of Module Functions | Patients believed that the functions of some modules could be further optimized. | “At the moment, monitoring tools can only analyze trends of general distortion area, but can't tell me about changes in how distorted things are.” (Patient 18) |
| **4. Strong Willingness for Continued Use** | 4.1 Hope to Continue Using for Condition Monitoring | Patients expressed their willingness to continue using the GridMacuScan. | “I'm hoping to carry on using this monitoring tool because it's beneficial for keeping track of how my condition is changing, and it provides a relatively objective assessment.” (Patient 7) |
